# Assessment of Body Composition in Health and Disease Using Bioelectrical Impedance Analysis (BIA) and Dual Energy X-Ray Absorptiometry (DXA): A Critical Overview

**DOI:** 10.1155/2019/3548284

**Published:** 2019-05-29

**Authors:** Maurizio Marra, Rosa Sammarco, Antonino De Lorenzo, Ferdinando Iellamo, Mario Siervo, Angelo Pietrobelli, Lorenzo Maria Donini, Lidia Santarpia, Mauro Cataldi, Fabrizio Pasanisi, Franco Contaldo

**Affiliations:** ^1^Department of Clinical Medicine and Surgery, University Federico II, Naples, Italy; ^2^Department of Biomedicine and Prevention, Division of Clinical Nutrition and Nutrigenomic, University of Rome “Tor Vergata”, Italy; ^3^Department of Clinical Science and Translational Medicine and School of Sports Medicine, University Tor Vergata, Rome, Italy; ^4^Scientific Institute of Research and Scientific Institute of Research and Care, San Raffaele, Pisana, Italy; ^5^Human Nutrition Research Centre, Institute of Cellular Medicine and Newcastle University Institute for Ageing, Newcastle University, Biomedical Research Building, Campus for Ageing and Vitality, Newcastle on Tyne, UK; ^6^Pediatric Unit, Verona University Medical School, Verona, Italy; ^7^Sapienza University of Rome, Experimental Medicine Department, Medical Pathophysiology, Food Science and Endocrinology Section, Food Science and Human Nutrition Research Unit, Rome, Italy; ^8^Division of Pharmacology, Department of Neuroscience, Reproductive and Odontostomatologic Sciences, Federico II University of Naples, Naples, Italy; ^9^Interuniversity Centre for Obesity and Eating Disorders (CISRODCA), Federico II University of Naples, Italy

## Abstract

The measurement of body composition (BC) represents a valuable tool to assess nutritional status in health and disease. The most used methods to evaluate BC in the clinical practice are based on bicompartment models and measure, directly or indirectly, fat mass (FM) and fat-free mass (FFM). Bioelectrical impedance analysis (BIA) and dual energy X-ray absorptiometry (DXA) (nowadays considered as the reference technique in clinical practice) are extensively used in epidemiological (mainly BIA) and clinical (mainly DXA) settings to evaluate BC. DXA is primarily used for the measurements of bone mineral content (BMC) and density to assess bone health and diagnose osteoporosis in defined anatomical regions (femur and spine). However, total body DXA scans are used to derive a three-compartment BC model, including BMC, FM, and FFM. Both these methods feature some limitations: the accuracy of BIA measurements is reduced when specific predictive equations and standardized measurement protocols are not utilized whereas the limitations of DXA are the safety of repeated measurements (no more than two body scans per year are currently advised), cost, and technical expertise. This review aims to provide useful insights mostly into the use of BC methods in prevention and clinical practice (ambulatory or bedridden patients). We believe that it will stimulate a discussion on the topic and reinvigorate the crucial role of BC evaluation in diagnostic and clinical investigation protocols.

## 1. Introduction

The human body comprises more than thirty measurable components [1]. A direct in vivo measurement of body components is currently not possible; consequently, indirect methods and models have been developed to do that. Within this framework, the World Health Organization (WHO) defines “*nutritional status*” as the condition of the body, resulting from the balance of intake, absorption, and utilization of nutrients interacting with individual physiological and pathological status.

The most frequently applied model to evaluate body composition (BC) in clinical practice and epidemiology splits the body into fat mass (FM) and fat-free mass (FFM), i.e., the bicompartmental model. FM indicates the water-free body component; the remaining body components (skeletal muscle, internal organs, and interstitial fat tissue) are included in the FFM. The most accurate methods to measure FM and FFM according to the bicompartment model are densitometry (underwater weighing), hydrometry (deuterium dilution), Echo-MRI, and total body potassium (TBK) counting. However, these methods are characterized by complex measurement protocols and require specialized expertise and costly equipment, making their application in clinical settings limited.

Bioimpedance analysis (BIA) is a widely used method to evaluate BC for both epidemiological and clinical purposes; it measures the electrical properties of body tissue and estimates BC parameters as total body water (TBW) and FFM BC parameters (see methods).

BIA is a noninvasive, low cost, and reliable method for BC assessment in clinical and nonclinical settings. The basic principle of the BIA technique is that the transit time of a low-voltage electric current through the body depends on BC characteristics [2]. However, this methodology has limitations due to the chemical composition of FFM (i.e., water, proteins, glycogen, and minerals) because of considerable inter- and intraindividual variability as a consequence of changes in FFM occurring with growth, maturation, ageing, and disease states [3].

Dual energy X-ray absorptiometry (DXA) is the current reference method for the assessment of BC, mainly because it provides accurate estimates of bone mineral, fat, and lean soft tissue (the so called three-compartment model) [4]. DXA utilizes low-emission X-rays to measure the attenuation of incident X-ray beams when they pass through body tissues (high attenuation for bone and low attenuation for fat).

The assessment of bone health to establish diagnosis of osteoporosis and fracture risk requires DXA for evaluating bone mineral density (BMD) in selected anatomical regions of interest (e.g., spine and femur). In addition, DXA is capable of providing estimates of visceral fat using validated predictive algorithms [5] and furnishes a measure of truncal fat mass, which has been found to be predictive of disease risk [6].

This review aims to summarize the scientific background of BIA and DXA and to furnish a comprehensive overview of their theoretical/technical concepts and application in bedridden and ambulatory patients and the information they can provide on drug pharmacokinetics.

## 2. Assessment of BC by BIA

BIA measures the electrical properties of body tissues and represents a useful approach for estimating body composition parameters such as TBW and FFM. In the bicompartment model, the human body is composed of FFM, which includes, under physiological conditions, the following components: bone mineral content (≈7%), extracellular water (≈29%), intracellular water (≈44%), and visceral protein (=20%). BIA estimation of body composition is based on body fluid volume measurement using BIA resistance value [2, 7, 8].

Bioelectrical impedance, or bioimpedance (*Z*, Ω), is defined as the opposition of a conductor to the flow of an alternating electrical current applied to it. Bioimpedance varies with tissue composition as well as with the frequency of the applied current. Bioimpedance is a complex parameter derived from the vector relationship between resistance (*R*, Ω), which arises from intracellular and extracellular fluids, and reactance (*X*_c_, Ω), which is related to the capacitance of the cell membrane [7]. Although the human body is not a uniform cylinder, an empirical relationship can be established between the ratio height^2^/*R* (cm^2^/Ω 50 kHz), defined as bioimpedance index (BI) measured at 50 kHz, and the volume of TBW, approximately 73% of FFM in healthy individuals.

Single-Frequency-BIA (SF-BIA), generally at 50 kHz, is passed between surface electrodes placed on hand and foot. Some BIA devices use other electrode placements, such as foot-to-foot or hand-to-hand electrode (Bipedal BIA). Many studies have compared multifrequency hand-to-foot (HF-BIA) and foot-to-foot (FF-BIA) bioimpedance analysis in order to assess differences in FFM values in populations with a wide range of body mass index (BMI) [9, 10] and they found that FF-BIA gives lowest values of FFM in overweight and obese subjects, also if compared with the results of the DXA [11]. In clinical practice, BIA allows monitoring of body fluids (extracellular/intracellular ratio) and therefore patients' nutritional status, in the short time and long time [12, 13].

### 2.1. Phase Angle

**Figure 1:**
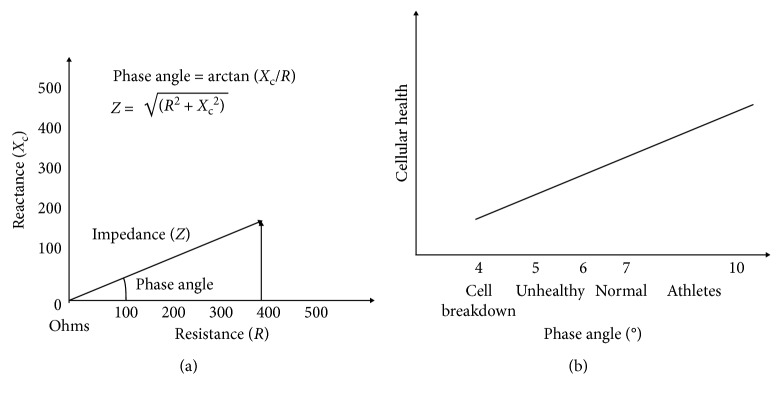
Phase angle.

The phase angle, or PA ((*R*/*X*_c_) × (180/*π*)), expressed in *degrees*) reflects the ratio between intra- and extracellular water. It may be affected by nutritional and hydration status [2] (Figure 1). In healthy subjects, PA ranges between 6° and 7° [14], and in athletes it may reach 8.5° [15]. Low PA (<5°) indicates the loss of cellular integrity [16–18]. The PA appears to be a more sensitive indicator of nutritional status compared to impedance since it is closely associated with cellular integrity [19–22].

### 2.2. Multifreqency BIA and BIA Spectroscopy

**Figure 2:**
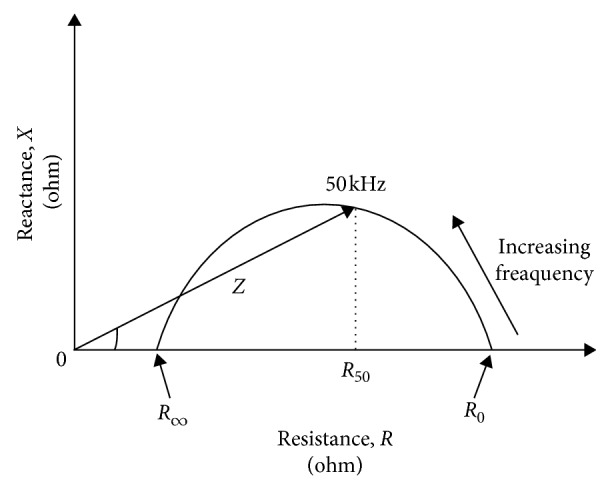
Bioimpedance spectroscopy (BIS) variation of impedance with frequency. Resistances extrapolated at zero (*R*_e_) and infinite (*R*_∞_) frequencies.

BIA can be performed using simultaneously electrical current with different frequencies. The application of more than two frequencies, ranging from low (1 kHz) to high (500 kHz) frequencies, allows the measurement of TBW, FFM, FM, and ICW and ECW compartments. At low frequencies (1–5 kHz), the electric current does not penetrate the cell membrane, and therefore it is assumed that the current passes through the extracellular fluid. Conversely, at higher frequencies (>50 kHz), the current passes through the cell membranes and it is associated with both intracellular and extracellular fluid compartments [23–25]. Frequencies higher than 100 kHz do not improve the accuracy of body composition estimation (Figure 2).

Bioimpedance spectroscopy (BIS) differs in the underlying, theoretical basis from the more commonly applied single-frequency BIA, because it does not require the use of statistically derived, population-specific prediction equations. One of the main advantages of the BIS is its ability to differentiate between ECW and ICW. BIS has been found to be accurate for measuring changes in fluid volumes [26].

### 2.3. Bioelectrical Impedance Vector Analysis (BIVA)

**Figure 3:**
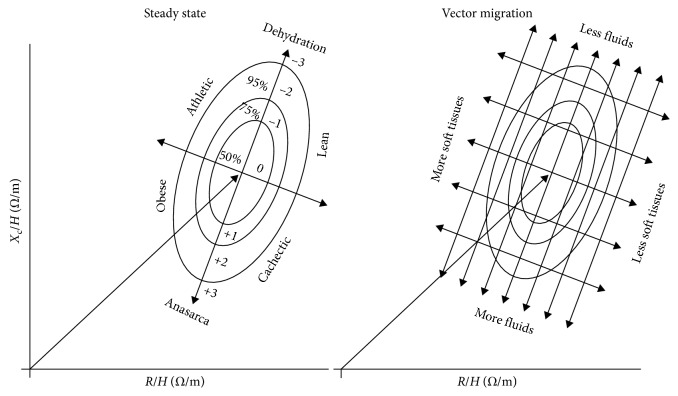
Bioelectrical impedance vector analysis (BIVA). *R* = resistance (ohm) measured at 50 kHz; *X*_c_ = reactance (ohm) measured at 50 kHz; *H* = height expressed in meters.

In the BIVA approach, introduced by Piccoli et al., [27] *R* and *X*_c_ (*R*-*X*_c_ graph), obtained at 50 kHz, are normalized to height (*R*/*ht* and *X*_c_/*ht*, respectively), and plotted as bivariate vectors (Figure 3). BIVA allows a direct assessment of body fluid volume through patterns of vector distribution on the *R*-*X*_c_ plane without the knowledge of the body weight. Reference tolerance ellipses (50, 75, and 95%) for the individual vector were previously calculated in the healthy population and specific patient populations. Bioelectrical vectors are analyzed by evaluating their position with respect to reference values (tolerance ellipses): a significant decrease in body hydration shifts the vector towards the upper pole of the ellipse major axis, whereas fluid retention moves it in the opposite direction. The vector shifts along the minor axis of the ellipse according to individual soft tissue body cell mass, shifting on the left side with more cell mass.

### 2.4. Assessment of Body Composition by Dual Energy X-Ray Absorptiometry (DXA)

Among different methods of body composition measurements, DXA provides whole body and regional estimates of three main components: FM, lean body mass (LBM), and bone mineral content (BMC). Several options are available as the first choice to investigate visceral fat, such as magnetic resonance imaging (MRI) or computer tomography (CT) scanning, because they provide a quantitative and qualitative assessment of visceral (pre- and postperitoneal) and subcutaneous (superficial and deep) adipose tissue [28,29]. However, costs, technical staff and expertise, contraindications, and accessibility to these methods are important limitations. Therefore, DXA is also used to investigate visceral fat.

DXA uses a source that generates X-rays, a detector, and an interface with a computer system for imaging the scanned areas of interest. The effective radiation doses involved are small (1–7 *μ*Sv), making the technique widely applicable. Due to DXA's advantages in terms of accuracy, simplicity, availability, and relatively low expense as compared to procedures like TBK, MRI or CT IMAGING, and low radiation exposure, DXA measurement is becoming increasingly important, emerging as reference assessment technique also in muscle mass evaluation [30]. DXA systems are practical, require no active subject involvement, and impose minimal risk [20, 31, 32]. Radiation exposure from a whole body DXA scan is equivalent to between 1 and 10% of a chest X-ray [20]. Moreover, unlike most other body composition methods that are designed to quantitate a single whole body component, DXA allows quantification of multiple whole body and regional components. As a result, DXA is gaining international acceptance as a body composition reference method [33], particularly in severe malnutrition and overweight/obesity.

### 2.5. Clinical Indications to BIA Utilization

Being a noninvasive method, BIA allows to follow body composition modifications in time, for example, in case of weight loss during acute or chronic diseases or, on the contrary, during weight gain, offering the possibility to have a prognostic forecast [34].

Anyway, there are several factors that can affect the BIA results, such as nonstandardization of body position, previous physical exercise, and food or fluid intake [35]. Also, different predictive equations have been developed to estimate TBW and FFM which include several parameters such as sex, age, and body weight. These predictive equations are generally population-specific and device-specific and can be useful only in individuals with the same characteristics of the reference population and with a physiological hydration status [2].

In addition, pathological conditions could modify the individual's hydration level (dehydration/edema). Hence, existing equations for FFM could not be used, in as much as they do not make a distinction between the amount of intracellular and extracellular water. The development and validation of specific equations is mandatory and should be the focus of future studies.

Regarding PA, it is a useful parameter in clinical practice as it allows identification and monitoring of patients at risk of impaired nutritional status and decreased survival, such as HIV/AIDS, cancer, anorexia, liver cirrhosis, hemodialysis, and pulmonary disease geriatric and surgical patients [21, 36–40].

Few studies have also addressed the possibility to apply PA in Sport Medicine to evaluate physical performance [41–43]. Silva et al. [32] described a positive correlation between handgrip strength and PA in elite judo athletes during a competition. Recently, Marra et al. [33] showed in a team of elite endurance cyclists, evaluated during their participation to a tournament-cycling race (Giro d'Italia), a significant and progressive reduction of PA. The reduction of the PA suggests a loss of intracellular water (ICW), which could be explained by the long-term competition and continuous vigorous exercise [44]. That study [44] showed that PA is a useful method for monitoring body composition and for obtaining information on the cell integrity, even if its relationship with sports performance is not readily evident. For this reason, in the future, it is advisable to conduct studies in elite athletes to verify the link between the PA and muscle strength and performance.

Despite the close correlation between nutritional status and phase angle, however, not all studies found the phase angle a reliable indicator of disease-related malnutrition. This led to the use of BIVA approach as an alternative tool to assess and monitor patients' hydration and nutrition status in several pathologic conditions, such as hemodialysis [45] or ambulatory peritoneal dialysis [46], liver cirrhosis [47], critically ill [48], and obese patients with stable and changing weight [49], because of its independence from regression equations in the calculation of lean body mass and fat mass and body weight.

In such a way, BIVA enables a more detailed understanding of hydration status and cell mass compared to phase angle alone. Since phase angle is calculated from reactance and resistance, different positions of the vector in the *R-X*_c_ graph can theoretically produce identical phase angles (Figure 3). Differentiation between obese (high phase angle, short vector) and athletic subjects (high phase angle and long vector) is consequently possible by BIVA just as discrimination between cachectic (low phase angle and long vector) and lean subjects (normal phase angle and long vector).

In conclusion, bioelectrical phase angle and BIVA represent a clinical approach to body composition, free from prediction equations-inherent errors and assumptions, although quantities of body compartments are not measured.

## 3. Clinical Indications to Use DXA

DXA is routinely used in clinical practice for the measurement of bone mineral tissue, allowing the diagnosis and the follow-up of osteoporosis, a potentially high-risk condition characterized by malabsorption, malnutrition, and long-term corticosteroid therapies, frequently observed in post menopause and in several chronic diseases.

The use of DXA for the assessment of body composition in daily clinical practice should be extended to overweight/obese patients in order to better evaluate their long-term cardiovascular and oncologic risk related to excessive adiposity.

BMI changes determined at individual level do not distinguish between increased body weight due to fat or nonfat mass. Indeed, WHO has defined BMI a good measure of adiposity at the population level, but a “surrogate” measure of adiposity at the individual level [50]. DXA measures excess adiposity with more accuracy than BMI, but, although promising, it is premature to recommend its routine use for the diagnosis of obesity because there have been few clear statements regarding its clinical indication for body composition assessment in patients outside the research setting [51]. However, DXA could be used to monitor changes in lean and fat tissues in obese subjects undergoing major weight losses, such as after bariatric surgery [52, 53]. In this condition, body weight might not change, but body composition might change during weight loss interventions. DXA allows to quantify total fat and lean soft tissue and also truncal and visceral fat [52], which are useful in the evaluation of cardiometabolic risk [54, 55]. Therefore, DXA may represent a method for clinical assessment of weight changes and/or training programs on fat and FFM compartments [51]. DXA analysis can also be used in patients with sarcopenia [30, 51]. This condition involves a decreased skeletal muscle mass and strength, and it is usually described in the elderly. Similarly to obesity, it is considered a risk factor for metabolic disease [56]. When sarcopenia and obesity occur concomitantly in an individual, the condition is referred to as sarcopenic obesity (SO) [57].

Using DXA, we could also acquire information on the three compartments (lean, fat, and bone) of the body, and four regions (i.e., head, trunk, arms, and legs) so as to obtain information on the efficacy of treatment in osteoporosis and other clinical conditions related to bone turnover.

Other examples of clinical indications to DXA are the following:

### 3.1. Pediatric Age

Body composition analysis in children provides a window into the complex changes that occur throughout childhood and gives the opportunity for understanding metabolic and physiological correlations [50, 51, 58]. DXA has the ability to evaluate nutritional status and growth disorders by analyzing the individual compartments of the body, thus offering the opportunity for studying skeletal maturation and mineral homeostasis in relation to environmental and/or pathological factors involved in the development [59–62].

### 3.2. Patients with HIV

DXA total body composition with regional analysis can be used in HIV patients to assess fat distribution in those using antiretroviral agents who are at risk of lipoatrophy [51]. DXA allows to detect the individual and independent effects of antiretroviral agents on peripheral (arm and leg) and central (trunk) fat. DXA has been demonstrated to be a highly sensitive and consistently reliable technique for detecting changes in fat distribution over a relatively short period (e.g., months) before clinically apparent lipodystrophy develops [51, 58].

### 3.3. Patients Candidate or Treated with Bariatric Surgery

DXA can be used in obese subjects undergoing bariatric surgery in order to monitor lean and fat mass changes. Repeat scans could be done at 3 months after bariatric surgery. Early detection of lean soft tissue decline during weight loss may prompt clinical recommendations for increasing physical exercise and more appropriate dietary advice [51, 63, 64], even though practical considerations limit the use of DXA in severely obese subjects.

### 3.4. Safety of DXA

There are no contraindications to the use of DXA in the clinical practice with the exception of pregnancy [65]. However, being a radiological procedure, DXA should be performed no more than twice per year, which is comparable to the exposure to an intercontinental flight, thus not requiring strict monitoring, at least in some patients [51].

## 4. Body Composition and Pharmacokinetics: A Window of Opportunities for Research and Therapeutics

There is still scant awareness on the issue that responses to drugs can be affected by changes in body composition. Even though obesity and cachexia, at the extremes, may interfere with drug pharmacokinetics and pharmacodynamics at multiple levels, the most relevant effects are on drug distribution, i.e., on the diffusion of drugs from the blood to the tissues [66–68]. Given that the total amount of a drug that moves from the blood into its distribution compartment (mainly fat mass for lipophilic drugs and fat-free mass for hydrophilic drugs) depends on the size of the compartment, drug distribution will be affected by body composition status. When a drug is administered to a patient with its relative distribution compartment(s) larger than that of normal, its peak concentration in plasma will be lower and the time for its disappearance from blood longer than normal, leading to smaller but longer pharmacological effects [67, 68].

Conversely, higher peak concentrations and shorter persistence in plasma are expected when its distribution compartment is smaller than normal, suggesting that, in these conditions, toxicity could be higher even in the setting of a lower clinical efficacy. The pharmacokinetic consequences of the expansion of drug distribution compartments have been studied in more details in general anesthesia in obese patients [68, 69]. Moreover, it has been repeatedly suggested that underdosing drug could be a very common problem in obese patients [70–72] and strategies for dose corrections in morbid obesity have been established [68, 73, 74]. However, the information for several classes of drugs in obesity is still very limited, and strong efforts are needed to address this issue.

In addition, until recently, little attention has been paid to the effects of the decrease in fat and/or fat-free mass on the pharmacokinetics of drugs in sarcopenic conditions, with the exception of few studies performed in selected pathologic conditions such as AIDS [75]. The interest on this issue boosted in the recent years after the publication of a series of influential papers showing that the dose-dependent toxicity of hydrophilic antineoplastic drugs such as 5-FU or capecitabine is higher in sarcopenic patients and inversely related with psoas muscle surface area measured by CT scan at the level of L3 [76]. This observation fits well with the evidence that FFM and, especially skeletal muscle mass, represents the main distribution compartment for these drugs [68]. The issue of drug distribution in muscles and its consequences in neoplastic patients with sarcopenia is further complicated by the evidence that some transduction therapy agents, such as sorafenib, may reduce muscle mass by a direct action [77]. This suggests potential, new, and unexpected interactions between different combination chemotherapy protocols with drugs that directly affect the size of the distribution compartments. Researches specifically focused on dose adjustments of drugs, according to body composition characteristics, are warranted for a precision, personalized therapy.

## 5. Future Directions

This review highlighted the relevance of body composition assessment and monitoring by BIA and DXA in the evaluation of nutritional status in several pathological conditions. However, for a wider clinical application, some issues related to these techniques should be addressed.

Future investigations on BIA could include the following:

- Improving validation of BIA equations according to age, sex, and ethnicity
- Developing specific equations for under or overhydrated patients
- Developing PA prognostic/survival predictive values in pathological conditions
- Accurate validation of MF-BIA, segmental BIA, and BIS in conditions of body fluid abnormalities (heart, liver, kidney diseases, etc.)

For DXA, future developments could be the following:

- Individuating factors that affect the accuracy of the methods, such as subject's body shape and size, calibration procedures, software version, and instrumental models
- Advanced analysis techniques that significantly reduce the impact of motion artifacts on infant DXA scans
- Highly standardized and reproducible patient positioning and image analysis procedures to accurately measure axial, appendicular, and segmental regions of interest
- Assessing how changes in fat distribution affect the accuracy of estimates/measurements, in as much as an estimated body composition by DXA changes with age, exercise, and diet

Finally, future studies appear mandatory to better understand the relationship between pharmacokinetics and pharmacodynamics of different drugs and BC in different nutritional states.
